# Disposal practices for unused and expired medications: pilot data from three cities in three countries

**DOI:** 10.3205/hta000133

**Published:** 2022-03-07

**Authors:** Khalid M Kamal, Marco Chiumente, Sari Nakagawa, Vincent Giannetti, Taylor Marlin

**Affiliations:** 1 West Virginia University School of Pharmacy, Departement of Pharmaceutical Systems and Policy, Morgantown, USA; 2 SIFaCT-Italian Society of Clinical Pharmacy and Therapeutics, Milan, Italy; 3 Kobe Gakuin University, Faculty of Pharmaceuticals Sciences, Kobe, Japan; 4 Duquesne University School of Pharmacy, Divison of Pharmaceutical, Administrative and Social Sciences, Pittsburgh, USA; 5 Duquesne University School of Pharmacy, Pittsburgh, USA

**Keywords:** expired medications, unused medications, pharmacy, Japan, Italy, United States

## Abstract

**Objective:** To collect pilot data on medication disposal practices of unused and expired medications from three cities in three countries.

**Methods:** A cross-sectional survey was conducted in Pittsburgh, United States (US); Turin, Italy; and Kobe, Japan. A convenience sampling was utilized through drug take-back programs in Pittsburgh, US; pharmacy customers in Turin, Italy; and pharmacy students and family members in Kobe, Japan. Descriptive analysis was conducted to assess medications disposal practices including attitudes and beliefs of respondents.

**Results:** The sample included 342 respondents [99 (Pittsburgh, US); 168 (Turin, Italy); and 75 (Kobe, Japan)]. The mean unused and expired medications per patient for Pittsburgh, US was (1.60±2.30 and 0.51±1.54); Turin, Italy (1.69±1.86 and 0.49±1.22) and Kobe, Japan (6.69Â±8.78 and 0.84±2.26). The major reason for unused medications in Pittsburgh, US (31.3%) was “Medication was as needed”; in Turin, Italy (28.0%) “No longer suffer from the condition”; and in Kobe, Japan (54.7%) “No longer suffer from the condition”. The most common reason for expired medications was “No longer suffer from the condition” (Pittsburgh, US 17.2%; Turin, Italy 15.5%; Kobe, Japan 12.0%). The disposal method in Pittsburgh, US was disposing in the toilet (35.4%); returned to the pharmacy in Turin, Italy (51.2%); and disposed the original container in the trash in Kobe, Japan (82.7%).

**Conclusions:** There is a need for counseling protocols regarding proper disposal, which can lead to better adherence, reduction of prescription drug abuse, and less environmental hazards due to improper disposal of prescription medications.

## Introduction

Physicians, pharmacists, and patients handle or use prescription medications on a daily basis, but there seems to be a gap on how to safely and effectively dispose unused and expired medications. Unused medications can be defined as non-expired medications once prescribed or recommended by a healthcare professional that are no longer in use. Expired medications are medications that are beyond their ‘use-by’ date. Both unused and expired medications can be considered unsuitable for therapeutic use because the patient either has no medical need for the medication or the medication is not guaranteed to be efficacious. There is evidence to show that improper disposal of unused and expired medications results in significant increase in medication abuse and misuse resulting in increased morbidity, mortality and healthcare costs. In addition to adversely affecting human health, the practice of improper disposal poses acute exposure risks to wildlife through contamination of the environment [1].

The United States (US) Food and Drug Administration (FDA) recommends either flushing medications or disposing them in the household trash [2], [3]. There is a national drug take-back day sponsored by the Drug Enforcement Administration (DEA). Typically, the events are held every April and October at community and hospital pharmacies across the nation. Some major chain pharmacies in the US have also recently installed medication disposal machines in their stores [4]. Other disposal methods available include disposal packets, which are dispensed upon request at community pharmacies. These disposal packets contain a powder that can render medications useless and unavailable to ensure safe disposal [5]. Unlike the US, Italy adheres to a national regulatory framework and drug disposal is managed at local levels. Japan has a national law that regulates the release of pharmaceuticals into the environment. However, Japan is still in the preliminary phase in terms of regulations when compared to Italy but its take-back schemes are voluntary and similar to those in the US. 

### Disposal practices in the United States

A few studies have explored drug disposal practices in the US. A randomized controlled trial study showed that more than half of the patients (54%) had unused opioid analgesics after surgery and behavioral intervention such as directing patients to drug disposal programs, led to a 22% increase in the number of patients who reported disposing of or intending to dispose of their medications [6]. Another study reported that people exposed to media encouraging proper drug disposal practices were twice as likely to prefer appropriate disposal for their medications than those who were not exposed [7]. A study conducted among university students showed that 77% of them did not store their medications in a locked or safe place and 28% of students witnessed sharing of prescription medications among peers [8]. Another study reported that 65.4% of patients surveyed keep and maintain their opioid medications even when not actively treating pain [9].

An additional factor to consider is that patients improperly handle or share their medications due to possessing an excessive amount of medications. One study examined the extent, amount, type, cost, and reasons for unused medications in US households. Surveys administered at 20 drug disposal events showed that two-thirds of returned prescriptions (for tablet, capsule, and liquid dosage forms) had more than half the original quantity remaining [10]. The results showed that two-thirds of the returned medications were expired, 25% of them were discontinued by a physician, and 18% of them were discontinued by the patient once they were feeling better. It was also noted that 71% of patients chose a pharmacy as their preferred medication disposal location. These results are significant because excessive medication wastage is estimated to cost $2.4 billion among elderly populations and $5.4 billion among US adults [10]. In addition to the substantial economic impact, environmental concerns have also been raised concerning the improper disposal of unused and expired medications including the specific effects of antibiotics, estrogens and antidepressant medications upon wildlife and possible effects upon human health by continued exposure. A study conducted by the US FDA shows that between 2004 and 2014 there has been a 742%, 564%, and 122% increase in environmental concentrations of buprenorphine, naloxone, and naltrexone, respectively. These increases can be attributed to increased clinical use [11]. There is limited data on other medications being flushed and subsequently permeating the water supply. Despite the lack of studies on the topic, many counties across the US have “don’t flush” programs. These programs encourage households to not introduce medications to the water supply in order to decrease water pollution [12].

### Disposal practices in Italy

The National Health Service, which is a public system that includes universal health care, was established in Italy with Law 833/1978, enacted to comply with Article 32 of the Italian constitution which enshrines the right to health for all individuals. This system is supported by general taxation and direct revenues. It is made up of 21 regional health services that ensures healthcare services for all citizens [13], [14]. The entire delivery of pharmaceutical products, even if managed by both private and public companies, is a responsibility of the regional health service.

Regarding the management of expired drugs, the regions must comply with all national regulations concerning the different waste categories. Italy adheres to European waste management standards, according to which drugs are classified as hazardous urban waste with some exceptions such as those that pose at a risk of infection or are radioactive. Regarding the drugs used outside the hospitals, disposal must be carried out through special containers located near retail pharmacies or in special places made available by the municipalities in accordance with regulatory bodies. The containers of expired or unusable drugs are managed by private companies under the control of the regions.

The pharmaceutical products, disposed in the containers by the citizens, are taken to special recycling sites and incinerated to reduce the potential harmful effect on the environment. Although the system is efficient and tested, occasional pollution levels of medication have been found in water reserves [15], [16]. Several studies are investigating methods to eliminate antibiotics, pesticides and other drugs through wastewater treatment. Similar to the US, the source of pollution is rarely due to improper disposal of waste by individual citizens, but rather to an inefficient management of wastewater from hospitals and various industries [17], [18].

### Disposal practices in Japan

The Waste Disposal Law is a Japanese law stipulating the processing and disposal method for waste matter and this includes medical waste. At pharmacies and medical facilities, expired or unused medical and pharmaceutical products are incinerated at a facility which conforms to the “Incineration Facility Construction and Maintenance Standards” (Regulation of the Waste Disposal Law). If no such facility exists, the pharmacy or medical facility takes responsibility for employing an approved industrial waste processor to dispose of the waste [19]. At the same time, medical waste in the home is classified as general household waste and under the Waste Disposal Law the municipality takes responsibility for its processing [20]. Concerning specific disposal methods recommended by municipalities when discarding of medical and pharmaceutical products, tablets and ointments are to be removed from their containers and wrapped in paper and liquid medications are to be absorbed into paper or cloth before being grouped with burnable trash. After this, these products are to be retrieved by the general waste processor employed by the municipality and incinerated at an incineration facility belonging to the local government. Although the contents of medical waste used and discarded at home are largely the same as those of waste generated by medical facilities, when it is discarded from the home, it is grouped with general waste which is processed and disposed of by the municipality; its handling is entrusted to the individual discarding the waste and its treatment may differ depending on the local government. As cases of in-home medical treatment continue to rise along with the aging population, it will be necessary to create rules that are shared across the country. 

Unused or expired medications can result in health, environmental, and economic hazards thus, it is necessary to ascertain the disposal practices and attitudes/beliefs of people with regards to their medication disposal practices. Medication wastage is an unnecessary burden on an already fiscally restrained healthcare system and it is important for healthcare providers and consumers to work together to find ways to control these costs. Although disposal practices are well known in the US, Italy, and Japan, there is a dearth of literature on these practices in Italy and Japan even with robust national policies and drug take-back programs. Thus, the objective of this study is to collect pilot data on medication disposal practices of unused and expired medications from three cities in three countries.

## Methods

### Survey design and participants

A cross-sectional survey was conducted in three cities of three countries — Pittsburgh, US; Turin, Italy; and Kobe, Japan. In the US, a convenience sampling was utilized through drug take-back programs in Pittsburgh and data was collected between the years 2015–2016. In Italy, a convenience sample of pharmacy customers were recruited from several community pharmacies in the city of Turin during the years 2015–2016. In Japan, a convenience sample of pharmacy students and their family members from Kobe Gakuin University were recruited in Kobe during the years 2012–2013 and 2015–2016. The pilot nature of the study allowed for a limited group of people to be recruited for the study, primarily those who were accessible to the authors in their respective countries. 

### Survey administration

In Pittsburgh, US; the survey was pretested in a small sample (n=5) and face validity was confirmed. The survey utilized in Pittsburgh, US was then translated in Italian and Japanese and back to Engish language and tested for accuracy by the authors (MC, SN) before being administered in cities of Turin and Kobe in Italy and Japan, respectively. The accuracy testing was conducted by authors in Italy and Japan and involved testing the equivalence of survey concepts and cultural concepts such as wordings or phrases. In doing so, the authors identified some minor changes that were made to the surveys in Italy and Japan and have been discussed in the next section.

### Survey questionnaire

The survey was developed in conjunction with a literature review and with discussion with key experts (including the authors) with knowledge on drug disposal practices. The survey determined the extent of unused and expired medications, attitudes and reasons regarding storing and disposal of unused and expired medications. The first and second sections of the questionnaire gathered information on the number of unused and expired medications along with the reasons for having these medications in the home. The third section gathered information on different medication disposal practices and included questions on the extent of physician and pharmacist counseling about disposal practices. In the fourth section, respondents rated on a 5-point scale in Pittsburgh, US (1=strongly disagree, 5=strongly agree) and a 4-point scale in Turin, Italy and Kobe Japan (1=strongly disagree, 4=strongly agree) their attitudes and beliefs regarding hazards related to medication disposal. The difference in scale was due to the fact that in both Italy and Japan, they do not prefer the “neither agree nor disagree” response option, which converted the 5-point Likert scale to 4-point. There were no other major changes in the survey across the three countries. The respondents were also asked if they were interested in acquiring more knowledge on the recommended methods of medication disposal. The last section collected demographic information — age, sex, race, ethnicity, marital status, education, annual household income and the services received from physicians, nurses and pharmacists in the last six month.

### Ethics statement

The study was approved by the Duquesne University Institutional Review Board. The study was performed according to the standards in the 1964 Declaration of Helsinki and its later amendments.

### Statistical analysis

Survey responses were tabulated and those with less than 50% completion were not included in the analyses. Country-wide data was separately analyzed and reported. Frequency distributions were determined for reasons for having unused and expired medications at home, different medication disposal practices, counseling received from physicians and pharmacists, and other demographic variables (e.g., sex, income, marital status, education). Descriptive statistics such as means and standard deviations (SDs), were used to report data such as age, unused and expired medications, provider services utilized, and attitude and beliefs responses. All the analyses were conducted using IBM SPSS Statistics (Version 25).

## Results

Overall, 342 respondents from Pittsburgh, US (99); Turin, Italy (168); and Kobe, Japan (75) completed the survey. The mean age of respondents were 49.77±18.93 years (Pittsburgh, US), 55.79±18.00 (Turin, Italy) and 23.15±8.42 years (Kobe, Japan). Majority of respondents were females in Pittsburgh, US (70.7%) and Turin, Italy (57.7%) and males in Kobe, Japan (48.0%) (Tab. 1 see Attachment 1).

Although the mean expired medications did not vary for Pittsburgh, US (0.5±1.54); Turin, Italy (0.49±1.22) and Kobe, Japan (0.84±2.26), differences were seen in the mean unused medications, especially in Kobe, Japan (6.69±8.78) compared to Pittsburgh, US (1.62±2.30) and Turin, Italy (1.69±1.86). The major reason for unused medications in Pittsburgh, US (31.3%) was “Medication was Pro Re Nata (PRN) or as needed”; in Turin, Italy (28.0%) was “No longer suffer from the condition”; and in Kobe, Japan (54.7%) was “No longer suffer from the condition.” The leading reason for expired medications in all the three cities in the three countries was “No longer suffer from the condition” (Pittsburgh, US 17.2%; Turin, Italy 15.5%; Kobe, Japan 12%) (Tab. 2 and Tab. 3 see Attachment 1). 

The general method of disposing medication varied widely across the three cities in the three countries. Disposing in the toilet was reported as the leading disposal method in Pittsburgh, US (35.4%) while in Turin, Italy, 51.2% reported returning the unused/expired medications to the pharmacy and in Kobe, Japan, 82.7% of the respondents reported disposing the unused/expired medications in their original containers in the trash. In terms of being counseled by a pharmacist on medication disposal, 51.8% of respondents reported positively in Turin, Italy with only 9.1% in Pittsburgh, US and 2.7% in Kobe, Japan. When queried if they ask their pharmacists about proper disposal of medications, 36.3% reported positively in Turin, Italy, followed by 15.2% in Pittsburgh, US and 1.3% in Kobe, Japan (Tab. 4 see Attachment 1). 

Regarding the respondents’ attitudes and beliefs about hazards related to medication disposal, the overall attitudes were favorable in Pittsburgh, US (4.42±0.76; scale of 1–5) and Kobe, Japan (3.19±0.66; scale of 1–4) while it was a little lower in Turin, Italy (2.51±0.56; scale 1–4). Specific question on the impact of disposal of medications with abuse potential that prevents drug abuse was highest for Pittsburgh (4.59±0.67) followed by Kobe (3.68±0.55) and Turin (2.54±0.52). Similar ratings were seen for questions on the impact on health care costs [Pittsburgh (4.50±0.69) followed by Kobe (3.03±0.68) and Turin (2.59±0.57)] and on environmental pollution [Pittsburgh (4.66±0.64) followed by Kobe (3.21±0.70) and Turin (2.67±0.54)]. When asked about their interest in acquiring more knowledge on the recommended methods of medication disposal, majority of respondents in all three cities [Pittsburgh (n=61; 70.1%), Turin (n=69; 92%)] and Kobe (n=100; 59.2%) expressed their interest. 

## Discussion

### Disposal practices

The study was conducted in three cities of three countries to collect pilot data on the similarities and differences in disposal practices of unused and expired medications, attitudes, and reasons regarding storing and disposal of unused and expired medications. Clearly, there were gaps seen in the delivery of pharmaceutical care services in the three countries. In Italy, in front (or behind) of every pharmacy, there is a basket where patients can dispose expired medications. This is a free service that reduces improper disposal and prevents abuse. Thus, the rate of unused/expired medication disposals at pharmacies were the highest in Turin, Italy (51.8%) compared to Pittsburgh, US (9.1%) or Kobe, Japan (2.7%). Similar to Italy, Japan also has a social insurance system but unlike Italy, Japan does not have a formal medication disposal program. Therefore, there are many people who did not show much concern for the inadvertent accumulation or proper disposal of unused or expired medications. In the US city of Pittsburgh, the results were mixed with a large proportion of respondents disposing medication improperly. Regarding unused medications in the US, the results of this study raise the question of PRN or “as needed” prescribing, where the timing of administering the prescription medication is left to the patient. Thus, there is a need for better patient monitoring and dispensing lower quantities of medication for PRN prescriptions, which could reduce waste and potential abuse [21]. For expired medications, the reason given by respondents in all three cities in the three countries was patients no longer suffered from the condition. This also raises the question of patient monitoring as clearer therapeutic end points and more frequent evaluation of patients by both the pharmacist and physician has the potential to reduce unused medications. This has both economic and patient safety benefits.

Less than 10% of the respondents in both Pittsburgh, US and Kobe, Japan reported being counseled on proper disposal. The majority of the sample in Pittsburgh and Kobe requested more information regarding disposal. Interestingly, respondents in Turin, Italy also requested more information on drug disposal although Italy has a good mechanism to handle unused/expired medications. Clearly, the pilot data from the three cities in the three countries suggests a need for pharmacists to provide counseling on drug disposal based on the respondents’ requests. In the US, efforts have been made to get the pharmacies involved in drug disposal, however, the US pharmacists seem to be reluctant to dispose medications unlike their Italian counterparts [22]. The response to the US DEA regulation that pharmacies could collect and destroy unused prescription drugs has been “insignificant” with only 1% reporting establishing drug disposal programs. A number of reasons have been cited as barriers to implementing these programs including the cost of collecting and disposing the medications, security risks involving collecting controlled substances, and some states in the US do not allow pharmacies to take back controlled substances. Even with these barriers, a number of pharmacies have installed drop-off boxes at their stores for safe disposal of medications. These pharmacies also sell DisposeRx and MedsAway packets, which can be used to mix their medications and dispose them safely [22].

### Attitudes and beliefs of respondents

The study data seem to suggest that effective disposal practices is independent of patient attitudes. Interestingly, even though Italy has a good system of medication disposal, respondents in the city of Turin had a lower attitude and beliefs regarding hazards related to medication disposal while it was higher in both Pittsburgh, US and Kobe, Japan. In the US, “Public Service Annoucements” style information sharing could be responsible for this difference in people’s attitudes and beliefs [23]. Broadcasting information may be effective in altering people’s attitudes because it is a non-confrontational way to encourage people to change their mindset and/or habits. In 2015, the Ministry of Health, Labour and Welfare in Japan released a report that estimated the yearly drug costs that could be cut due to leftover medication resulting from missed or incomplete doses at approximately 330 billion Yen (~$3 billion US dollars) [24]. Accompanying the aging population, there is also the issue of stockpiling of medical and pharmaceutical products by older adult patients with chronic disease [25].Under such circumstances, one countermeasure being carried out by pharmacies in regions nationwide is the “SETSUYAKU-BAG Campaign” which aims to cut back on wasted medicine. Launched in 2012, the “SETSUYAKU-BAG Campaign” is an initiative in which patients bring their leftover prescription medication to a pharmacy and a pharmacist confirms the amount leftover or the expiration date then contacts a physician to modify the prescription dose [26]. This campaign is based on activities such as the Brown Bag Review carried out in the US in the 1980s in which patients brought their multiple medications to a pharmacy in a brown paper bag and a pharmacist checked them for interactions before explaining their proper use. These movements ultimately reduce the burden of medication costs on patients thereby largely curtailing medical expenses. Concerning the appropriate disposal of unneeded medical and pharmaceutical products, it is expected that this movement will promote the appropriate disposal by pharmacists at local pharmacies, rather than in the home.

### Implications for clinical practice

Both physicians and pharmacists can play an important role in improving drug disposal practices and promoting the impact of such good practices on social, economic and environmental outcomes [27]. Majority of respondents from the three cities of the three countries reported that they no longer suffered from the condition for which the medication was prescribed. Thus, both physicians and pharmacists have a responsibility in reviewing medical histories and prescription medications including unused medications and counseling patients on correct disposal methods. In Pittsburgh, US, over 12.5% of respondents kept their medications for later use, a behavior that should be addressed by the providers. The exposure of unused or expired medications, especially to children, poses an intentional or accidental risk. From 2007 to 2016, there were 11.,275 children and adolescents (less than 19 years old) exposed to buprenorphine in a home setting [11]. Over 77% of these buprenorphine exposures among adolescents were defined as intentional, with 27.7% of exposures involving multiple substances [28]. Healthcare providers may lack the needed knowledge to properly counsel patients on medication disposal; any learning gaps should be addressed so that providers can counsel patients on why it is important to properly dispose of medications. 

Drug disposal programs seem to be able to increase safety among patients by encouraging patients to return their unused, expired, or discontinued medications to a pharmacy [29]. Potentially, pharmacies could gather data at these events and introduce cost-effective measures to decrease the amount of excess medication in a patient’s possession. Given the interest of respondents in brief counseling by pharmacists and pharmacies willing to take back expired and unused medications could significantly reduce the problem of unused and expired medications contaminating the environment or compromising population safety. As more pharmacies get involved in providing these services, there is a need for counseling protocols at a community pharmacy level regarding proper disposal, nonadherence, reduction of prescription drug abuse, and other factors that may reduce the extent of unused and expired medication storage. Further studies need to be conducted to assess the economic impact of unused and expired medications upon total prescription expenditures. 

### Study limitations

The study has all the limitations typically seen with survey methodology. As the study was done at a pilot in three cities, caution has to be exercised when extrapolating the study results to the three countries. Also, before drawing broad conclusions, the following limitations need to be considered. The survey results, even though collected using the same questionnaire, may not be directly comparable given the differences in social and health care systems, disease prevalence, population characteristics such socioeconomic status and education. The survey samples in these countries were different and so were the survey administration timelines. Although all the respondents were pharmacy customers, in Pittsburgh, US; the respondents were part of a drug take-back program where as in Kobe, Japan, they were pharmacy students and in Turin, Italy; they were general customers. Finally, a comprehensive linguistic and cross-cultural validation of the instrument translated in Italian and Japanese languages was not conducted. It is however clear from the study, that these countries need to make an investment in public health including increasing knowledge of the population regarding the appropriate use of drugs, correct disposal of waste by government or private organizations, and introducing civic education about drug disposal practices. The patient, who does not properly dispose of his/her own drugs, should not be labeled as guilty but we have to recognize that we are all part of a system that works when everyone shares the responsibility.

## Conclusions

The pilot study showed that majority of respondents in the three cities of the three countries expressed their interest in acquiring more knowledge on the recommended methods of medication disposal. Given the universal safety issue around inappropriate medication disposal, there is a need for counseling protocols regarding proper disposal, which can lead to better adherence, reduction of prescription drug abuse, and less environmental hazards due to prescription medications. The pilot study was able to identify population characteristics and logistical research issues in the three countries and will be vital in planning a proposed multi-country intervention on addressing people’s behavior related to disposal of expired and unused medications. 

## Notes

Reviewer comments see Attachment 2.

## Acknowledgements

Contributions by Mala Virginia Milkovich, Pharm.D., M.S. for assisting with the preparation and editing of this manuscript. The authors thank the Collegio Indipendente Subalpino Arti Farmaceutiche (CISAF) association for the support to the surveys collection in Italy.

## Conflict of interest

The authors declare that they have no competing interests.

## Supplementary Material

Table 1-4

Review comments
