# Supplementary material for: Disposal practices for unused and expired medications: pilot data from three cities in three countries
Source: GMS Health Innov Technol. 2022 Mar 7;16:Doc01. doi: 10.3205/hta000133 (PMC8953661; doi:10.3205/hta000133)
Supplement: Table 1-4 [file HInT-16-01-s-001.pdf]

Disposal practices for unused/expired medications, Table 1–4

Table 1: Demographic Characteristics of Respondents

	Pittsburgh, United States	Turin, Italy	Kobe, Japan
Sample Size	99	168	75
Mean Age in years	49.77±18.93	55.79±18.00	23.15±8.42
Gender	F (70, 70.7%); M (17, 17.2%)	F (97, 57.7 %); M (69, 41.1 %)	F (31, 41.3 %); M (36, 48.0 %)
Race	White (80, 80.8%), Black (4, 4.0%) Asian (2, 2.0%), Other (1, 1.0%)		
Marital Status	Single (31, 31.3%), Married (40, 40.4%), Divorced (9, 9.1%), Sepa (1, 1.0%)	Single (38, 22.6%); Married (110, 65.5 %), Divorced (8, 4.8%); Sepa (7, 4.2%)	Single (72, 96.0 %); Married (2, 2.7 %)
Education	Less than HS (4, 4.0%), HS/GED (12, 12.1%), Some college (22, 22.2%), College (22, 22.2%), Graduate (27,27.3%)	Less than HS (39, 23.4 %), HS/GED (83, 49.7 %), Some college (10, 6.0 %), College (15, 9.0 %), Graduate (20, 12.0 %)	High School graduation (70, 93.3 %); Graduation from University (4, 5.3 %)
Income	<$25000 (12, 12.1%), 25-50 (17, 17.1%), 50-75 (19, 19.2%), 75 (30, 30.3%)	<Euros25000 (48, 28.6 %), 25-50 (61, 36.3 %) 50-75 (31, 18.5 %), 75 (11, 6.5 %)	0 (34, 45.3 %); Less than 2 million Yen (3, 4.0 %); 2-4 Million Yen (4, 5.3 %); 4-6 Million Yen (4, 5.3%); 6 Million Yen or more (16, 21.3 %) [$1=Yen 120]
Last 6 months, services from Physician	3.12±2.86	3.19±3.48	2.78±3.04
Last 6 months, services from Pharmacists	3.81±4.25	4.94±8.52	1.81±2.54
Last 6 months, services from Nurse/ Physician Assistant	0.79±1.16	1.21±1.99	0.69±1.44

Attachment to: *Kamal KM, Chiumente M, Nakagawa S, Giannetti V, Marlin M. Disposal practices for unused and expired medications: Pilot data from three cities in three countries. GMS Health Innov Technol. 2022;16:Doc01. DOI: 10.3205/hta000133. Available from: https://www.egms.de/static/en/journals/hta/2022-16/hta000133.shtml*

Table 2: Respondents' Practices Related to Unused Medications

	Pittsburgh, United States	Turin, Italy	Kobe, Japan
Sample Size	99	168	75
Mean *Unused* Medications	1.60±2.30	1.69±1.86	6.69±8.78
Medications Prescribed to respondent	53 (53.5%)	81 (48.2%)	57 (76.0%)
Reasons for having *unused* prescription medications at home	Medication was 'take as needed'<br>31 (31.3%)	No longer suffer from the condition for which the medication was prescribed<br>47 (28.0%)	No longer suffer from the condition for which the medication was prescribed<br>41 (54.7 %)
	No longer suffer from the condition for which the medication was prescribed<br>29 (29.3%)	Medication was taken as needed<br>46 (27.4%)	Kept for later use<br>31 (41.3%)
	Switched to another medication<br>27 (27.3%)	Kept for later use<br>31 (18.5%)	Medication was taken as needed<br>20 (26.7%)
	Doctor discontinued medication<br>25 (25.3%)	Switched to another medication<br>25 (14.9%)	Forgot to take medications<br>15 (20.0%)
	Kept for later use<br>18 (18.2%)	Doctor discontinued medication<br>17 (10.1%)	Forgot to take stock of the medicine<br>5 (6.7%)
	Not aware of the proper method of disposal<br>15 (15.2%)	Experienced too many side effects<br>16 (9.5%)	Switched to another medication<br>4 (5.3%)
	Experienced too many side effects<br>15 (15.2%)	Medication was ineffective<br>9 (5.4%)	Medication was ineffective<br>4 (5.3%)
	Medication was ineffective<br>12 (12.1%)	Forgot to take stock of the medicine<br>12 (7.1%)	Not aware of the proper method of disposal<br>3 (4.0%)
	Other 9 (9.1%)	Forgot to take medications<br>3 (1.8%)	Experienced too many side effects<br>2 (2.7%)
	Forgot to take stock of the medicine<br>6 (6.0%)	Saved medication in case another family member might need it<br>3 (1.8%)	Saved medication in case another family member might need it<br>2 (2.7 %)
	Forgot to take medications<br>2 (2.0%)	Medication is expensive<br>-	Doctor discontinued medication<br>1 (1.3%)
	Medication is expensive<br>1 (1.0%)	Not aware of the proper method of disposal<br>-	Other (2 responses)<br>- I ask physician to prescribe extra since it's difficult for me to visit hospital frequently<br>- Physician prescribes extra medication
	Saved medication in case another family member might need it-	Other<br>-	Medication is expensive<br>-

Attachment to: *Kamal KM, Chiumente M, Nakagawa S, Giannetti V, Marlin M. Disposal practices for unused and expired medications: Pilot data from three cities in three countries. GMS Health Innov Technol. 2022;16:Doc01. DOI: 10.3205/hta000133. Available from: https://www.egms.de/static/en/journals/hta/2022-16/hta000133.shtml*

Table 3:Respondents’ Practices Related to Expired Medications

	Pittsburgh, United States	Turin, Italy	Kobe, Japan
Sample Size	99	168	75
Mean *Expired* Medications	0.51±1.54	0.49±1.22	0.84± 2.26
Medications Prescribed to respondent (expired)	16 (16.2%)	28 (16.7%)	16 (21.3%)
Reasons for having *expired* prescription medications at home	No longer suffer from the condition for which the medication was prescribed<br>17 (17.2%)<br><br>Medication was ‘take as needed’<br>13 (13.1%)<br><br>Doctor discontinued medication<br>12 (12.1%)<br><br>Not aware of the proper method of disposal<br>12 (12.1%)<br><br>Kept for later use<br>11 (11.1%)<br><br>Switched to another medication<br>11 (11.1%)<br><br>Forgot to take stock of the medicine<br>9 (9.0%)<br><br>Medication was ineffective<br>7 (7.0%)<br><br>Experienced too many side effects<br>7 (7.0%)<br><br>Other<br>3 (3.0%)<br><br>Forgot to take medications<br>2 (2.0%)<br><br>Medication is expensive<br>1 (1.0%)<br><br>Saved medication in case another family member might need it<br>-	No longer suffer from the condition for which the medication was prescribed<br>26 (15.5%)<br><br>Forgot to take stock of the medication<br>18 (10.7 %)<br><br>Medication was taken as needed<br>15 (8.9%)<br><br>Switched to another medication<br>10 (6.0%)<br><br>Doctor discontinued medication<br>3 (1.8%)<br><br>Forgot to take medications<br>3 (1.8%)<br><br>Experienced too many side effects<br>2 (1.2 %)<br><br>Kept for later use<br>1 (0.6 %)<br><br>Medication was ineffective<br>-<br><br>Medication is expensive<br>-<br><br>Not aware of proper method of disposal<br>-<br><br>Saved medication in case another family member might need it<br>-<br><br>Other<br>-	No longer suffer from the condition for which the medication was prescribes<br>9 (12.0%)<br><br>Kept for later use<br>5 (6.7%)<br><br>Forgot to take medications<br>4 (5.3%)<br><br>Not aware of proper method disposal<br>3 (4.0%)<br><br>Medication was ineffective<br>2 (2.7%)<br><br>Switched to another medication<br>2 (2.7%)<br><br>Doctor discontinued medication<br>1 (1.3%)<br><br>Medication was taken as needed<br>1 (1.3%)<br><br>Experienced too many side effects<br>1 (1.3%)<br><br>Medication is expensive<br>-<br><br>Forgot to take stock of the medication<br>-<br><br>Saved medication in case another family member need it<br>-<br><br>Other<br>-

Table 4: Methods Utilized for Disposing Unused and Expired Medications

	Pittsburgh, United States	Turin, Italy	Kobe, Japan
Sample Size	99	168	75
Methods used for disposing *expired* or *unused* medications	Flush medication in toilet<br>35 (35.4%)<br><br>Throw medication (in original container) in trash<br>34 (34.3%)<br><br>Return medication back to a take-back program<br>31 (31.3%)<br><br>Throw medication (not in original container) in trash<br>30 (30.0%)<br><br>Keep for later use<br>26 (26.3%)<br><br>Pour medication down the sink<br>9 (9.0%)<br><br>Pour in kitty litter or coffee grounds<br>7 (7.0%)<br><br>Return medication back to pharmacy<br>7 (7.0%)<br><br>Share medication with another person<br>4 (4.0%)<br><br>Other<br>4 (4.0%)<br><br>Sell medication<br>1 (1.0%)	Return medication back to pharmacy<br>86 (51.2%)<br><br>Return medication back to a take back program<br>58 (34.5%)<br><br>Throw medication (not in original container in trash)<br>27 (16.1%)<br><br>Keep for later use<br>12 (7.1%)<br><br>Other<br>7 (4.2%)<br><br>Flush medication in toilet<br>3 (1.8 %)<br><br>Throw medication (in original container in trash)<br>3 (1.8%)<br><br>Pour medication down the sink<br>2 (1.2%)<br><br>Share medication with another person<br>2 (1.2 %)<br><br>Pour in kitty litter or coffee grounds<br>-<br><br>Sell medication<br>-	Throw medication (in original container in trash)<br>62 (82.7%)<br><br>Return medication back to a take back program<br>43 (57.3 %)<br><br>Other<br>41 (54.7%)<br><br>Keep for later use<br>9 (12.0 %)<br><br>Throw medication (not in original container in trash)<br>9 (12.0%)<br><br>Pour medication down the sink<br>3 (4.0 %)<br><br>Flushed medication in toilet<br>1 (1.3%)<br><br>Sell medication<br>1 (1.3%)<br><br>Pour in kitty litter or coffee grounds<br>-<br><br>Return medication back to pharmacy<br>-<br><br>Share medication with another person<br>-
Do you check the medication expiration date?	74 (74.7%)	156 (92.9%)	24 (32.0%)
Have you ever been counseled by your physician on medication disposal?	2 (2.0%)	27 (16.1 %)	1 (1.3%)
Have you ever been counseled by your pharmacist on medication disposal?	9 (9.1%)	87 (51.8 %)	2 (2.7%)
Do you ask your pharmacist about proper disposal of your unused or expired medications?	15 (15.2%)	61 (36.3 %)	1 (1.3%)

Attachment to: *Kamal KM, Chiumente M, Nakagawa S, Giannetti V, Marlin M. Disposal practices for unused and expired medications: Pilot data from three cities in three countries. GMS Health Innov Technol. 2022;16:Doc01. DOI: 10.3205/hta000133. Available from: https://www.egms.de/static/en/journals/hta/2022-16/hta000133.shtml*
