# Supplementary material for: Disposal practices for unused and expired medications: pilot data from three cities in three countries
Source: GMS Health Innov Technol. 2022 Mar 7;16:Doc01. doi: 10.3205/hta000133 (PMC8953661; doi:10.3205/hta000133)
Supplement: Review comments [file HInT-16-01-s-002.pdf]

Disposal practices for unused/expired medications, Review comments

The review of your manuscript "DISPOSAL PRACTICES FOR UNUSED AND EXPIRED MEDICATIONS: PILOT DATA FROM THREE CITIES IN THREE COUNTRIES“ has been completed. The reviewers suggest that some changes should be integrated in the manuscript prior to release.

These are the reviewers’ specific comments:

Reviewer 1:
The authors provided a fine research on the disposal of medicines, a very interesting topic nowadays. The article gives valuable information. The introduction is clear, sets the scene sufficiently and classifies the own work into the available research. The methods are described sufficiently and understandable. The result section is also understandable and clear. The discussion is sufficient and suitable. The study limitations are described thoroughly. There are some small comments below. All in all a fine article recommended for publication with minor changes - It is recommended to formulate the research question end of the introduction in a single sentence. This would also clarify, that the aim is to collect pilot data, as it is also mentioned in the abstract - As it is classified as a pilot study, it would be interesting whether and for what research the pilot will be used? - Discussion: it should be discussed why flushing seems the main way of disposal in Pittsburgh, despite of the opposite recommendation of the FDA? - Limitations: it is mentioned, that there might be limitations due to "differences in social and health care systems, disease prevalence, population characteristics such socieoeconomic status and education". This is ok. but somewhat general, especially as if some differences can be seen clearly. It is recommended to refer to this a bit more in the discussion section. For example the different age of the respondents (US + Italy vs Japan) could have impact on the behaviour/results. What for? - Abstract results and also in results section: it might be difficult to understand the pure number of the mean given in abstract and results and therefore the relationship in between. It is recommended to explain - “Pro RE Nata (PRN). The article might be for interest for reader not being physicians of pharmacists used to Latin expressions. It is recommended to explain - Page 7 last paragraph, first sentence: “Regarding the respondents’…”. For better understanding the number referring the overall attitudes mentioned in this sentence should be given - Page 9, last sentence: double “that” -

Reviewer 2:
A general interest of discussion about different disposal practies for unused and expired medications is fully conclusive. Future research activities appear to make absolutely sense. Nevertheless, an improvement of the research methodology seems necessary for a follow-up study on a larger scale. Limitations, discussion and conclusion were provided in different sections. The research question is not clearly described. It is recommended to include the research question in the last sentence of the summary. It is also recommended to include the sample size calculation criteria (Which methods was used to calculate? How was the sample size been calculated?) as well as eligible criteria. After the pilot study it should be also considered to include a more comprehensive description of statistical methods that were used.

It should be emphasised more strongly that this is a pilot study in which not all aspects could be considered yet. Above all, the methodology should be given more consideration in a future primary study. International comparisons on this topic are difficult, but very interesting. It should be given more attention and this has been done with this article.

Attachment to: *Kamal KM, Chiumente M, Nakagawa S, Giannetti V, Marlin M. Disposal practices for unused and expired medications: Pilot data from three cities in three countries. GMS Health Innov Technol. 2022;16:Doc01. DOI: 10.3205/hta000133. Available from: https://www.egms.de/static/en/journals/hta/2022-16/hta000133.shtml*
